# Transmission of Novel Influenza A(H1N1) in Households with Post-Exposure Antiviral Prophylaxis

**DOI:** 10.1371/journal.pone.0011442

**Published:** 2010-07-07

**Authors:** Michiel van Boven, Tjibbe Donker, Mariken van der Lubben, Rianne B. van Gageldonk-Lafeber, Dennis E. te Beest, Marion Koopmans, Adam Meijer, Aura Timen, Corien Swaan, Anton Dalhuijsen, Susan Hahné, Anneke van den Hoek, Peter Teunis, Marianne A. B. van der Sande, Jacco Wallinga

**Affiliations:** 1 Centre for Infectious Disease Control, National Institute for Public Health and the Environment, Bilthoven, The Netherlands; 2 Department of Farm Animal Health, Faculty of Veterinary Medicine, Utrecht University, Utrecht, The Netherlands; 3 Unit for Infectious Disease Control, Public Health Service Hollands Midden, Leiden, The Netherlands; 4 Department of Infectious Diseases, Public Health Service Amsterdam, Amsterdam, The Netherlands; 5 Julius Center for Health Research and Primary Care, University Medical Center Utrecht, Utrecht, The Netherlands; Fred Hutchinson Cancer Research Center, United States of America

## Abstract

**Background:**

Despite impressive advances in our understanding of the biology of novel influenza A(H1N1) virus, little is as yet known about its transmission efficiency in close contact places such as households, schools, and workplaces. These are widely believed to be key in supporting propagating spread, and it is therefore of importance to assess the transmission levels of the virus in such settings.

**Methodology/Principal Findings:**

We estimate the transmissibility of novel influenza A(H1N1) in 47 households in the Netherlands using stochastic epidemic models. All households contained a laboratory confirmed index case, and antiviral drugs (oseltamivir) were given to both the index case and other households members within 24 hours after detection of the index case. Among the 109 household contacts there were 9 secondary infections in 7 households. The overall estimated secondary attack rate is low (0.075, 95%CI: 0.037–0.13). There is statistical evidence indicating that older persons are less susceptible to infection than younger persons (relative susceptibility of older persons: 0.11, 95%CI: 0.024–0.43. Notably, the secondary attack rate from an older to a younger person is 0.35 (95%CI: 0.14–0.61) when using an age classification of ≤12 versus >12 years, and 0.28 (95%CI: 0.12–0.50) when using an age classification of ≤18 versus >18 years.

**Conclusions/Significance:**

Our results indicate that the overall household transmission levels of novel influenza A(H1N1) in antiviral-treated households were low in the early stage of the epidemic. The relatively high rate of adult-to-child transmission indicates that control measures focused on this transmission route will be most effective in minimizing the total number of infections.

## Introduction

Recent studies have begun to unravel key epidemiological characteristics of novel influenza A(H1N1) virus, such as the incubation time, generation interval, and case fatality rate [1]–[6]. A major unknown is the infection probability per contact between an infected and a susceptible person, and how this probability depends on age and the use of antiviral drugs. Influenza is transmitted largely through close contacts, and the major locations where such transmission events take place are workplaces, schools, and households [7]–[9]. Of these, households provide the best defined setting and lend themselves naturally to study transmission rates.

Although there is a large body of literature on household studies for seasonal influenza, when a large proportion of the population is immune to infection [10]–[14], reports on transmission of novel influenza A virus within households remain scarce [4]–[5], [15]–[16]. Yet, such studies are vital to be able to tailor preventive household measures, not only because the characteristics of the novel influenza A virus may differ from seasonal influenza A viruses [17]–[19], but also because it is expected that for the novel influenza A virus a much larger fraction of the population has little or no pre-existing immunity.

Here we analyze detailed data from 47 households with a confirmed index case. During the study period antiviral drugs (oseltamivir) were provided therapeutically to confirmed infected cases and prophylactically to their household members. Such a policy has been predicted to reduce transmission to the extent that it may contain a pandemic at the start and to provide substantial benefit once a pandemic has taken off [7]–[8]. But as the timing of taking antiviral drugs depends on when the first infected case in a household has been detected, and the dose of antiviral drugs depends on age, it is a major question how influenza spreads in households that are provided with antiviral drugs. Our study shows that in this setting overall transmission efficiency is low, and that children are more susceptible to infection and less infectious than older individuals.

## Methods

### Case definition and case finding

From 29 April 2009 until 15 August 2009, novel influenza virus A(H1N1) infection was a notifiable disease in the Netherlands, requiring medical doctors and laboratories to report the patient to the Municipal Health Service when the disease is suspected or identified. Cases are defined as any person with one of the following clinical criteria: i) fever >38°C and signs and symptoms of acute respiratory infection, ii) pneumonia (severe respiratory illness), iii) death from an unexplained acute respiratory illness, meeting at least one of the following epidemiological criteria in the seven days before onset of the disease: 1) close contact to a confirmed case of novel influenza A(H1N1) virus infection while the case was ill, 2) travelling to an area where sustained human-to-human transmission of novel influenza virus A(H1N1) is documented, 3) working in a laboratory where samples of novel influenza A(H1N1) virus are tested (EC decision 2009/363/EC) [20].

In case of laboratory confirmation of novel influenza A(H1N1) contact tracing was performed by Municipal Health Services. Household and other close contacts of confirmed cases were tested for novel influenza virus A(H1N1) [20]. 47 index cases (36% male) and 109 household contacts (50% male) were enrolled in the study in the period between 29 April and 23 June. Household contacts were defined as persons living in the same residence as the index case.

Throat swabs were taken from all persons in the households, and analyzed using a general influenza A and a novel influenza virus A(H1N1) specific real-time RT-PCR [21]. Laboratory testing was performed by the National Influenza Centre in the Netherlands (Erasmus Medical Centre Rotterdam and National Institute for Public Health and the Environment Bilthoven).

Until 23 June, oseltamivir treatment was recommended for all laboratory confirmed cases and for their close contacts, regardless of symptoms. Index cases and household contacts were put on antiviral drug therapy within 24h after sampling if the test result was positive. There were no significant differences between households with and without secondary cases in the symptoms-to-sampling delay (mean: 1.7 vs 1.6 days). The average delay between the moment of onset of symptoms of the index case and his/her initial sampling was 1.4 days (number of cases: 47; range: −2 to 4 days; median: 1 day). The average delay between the moment of onset of symptoms of the index case and the sampling of the household contacts was 2.9 days (number of persons: 109; range: 1–7 days; median: 3 days), and between the moment of onset of symptoms of the secondary case and his/her sampling was 0.6 days (number of cases: 9; range: −1 to 4 days; median: 1 day). Persons that were found positive were usually sampled more than once. However, persons that were negative in the first test were not routinely tested for a second time.

All cases included in this study were from the period in which novel influenza A(H1N1) was a notifiable disease in the Netherlands (29 April 2009 until 15 August 2009). During this period both the diagnostic laboratories and the treating physicians were required by law to immediately report suspected and confirmed cases to the municipal health services. The municipal health services approached suspected and confirmed cases and their contacts for further investigation in the context of their task in source investigation. Hence, no approval from a medical ethical committee was required because novel influenza A(H1N1) was a notifiable disease, and the (anonymized) information used in this study was collected as part of the routine surveillance system. After 15 August, only hospitalised or deceased cases of novel influenza A(H1N1) remained notifiable, and contact tracing and routine antiviral treatment of cases and household members were halted.

### Statistical analysis

The analyses are based on the final size distribution of multitype stochastic SEIR models [13], [14], [22]. We classify persons as younger (type 1) and older (type 2), with a default age classification of ≤12 versus >12 years. The parameters *n_i_* and *a_i_* denote the initial number of susceptible and initially infected type 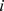 persons in a household, respectively (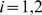). We assume that there are no persons that have prior immunity, so that total household size is given by *N = n_1_+n_2_+a_1_+a_2_*. The parameter *B_i_* denotes the probability that a type 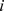 (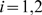) person escapes infection from outside the household. In this setting the final size distribution is determined by triangular equations for the ordered infection probabilities 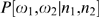 that can be solved recursively:
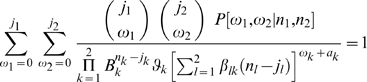
(1)(*j_1_ = 0,…*, *n_1_*, *j_2_ = 0,…*, *n_2_*) [13], [14], [22]. For given *n_i_* and *a_i_* the final size distribution is fully specified by the escape probabilities 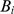, the Laplace transforms 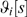 defining the probability distributions of the infectious periods, and the transmission parameters 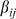 (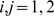) (see below for details). With equation (1) at hand the final size probabilities can be calculated recursively, starting with the probability that all persons escape infection (which is determined by 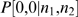 and can be calculated by taking *j_2_ = j_1_ = 0* in the above equation), and subsequently use 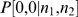 to calculate 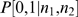 and 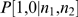, etcetera.

Since there was only limited transmission in the early stages of the epidemic and no epidemiological links between households could be found [20], we assume that households are independent. Hence, the likelihood function is given by the product of the likelihood contributions of the individual households. In practice, it is computationally more efficient to use the log-likelihood instead of the likelihood, and all calculations in this paper are based on the log-likelihood function. As a service to the reader we have included supporting information detailing the contributions of the individual households to the likelihood function in the specific case of an infectious period of fixed duration (Table S1).

A number of additional simplifications can be made because of the fact that there was only very little community transmission during the study period: no additional transmission from the outside into the household (*B_1_ = B_2_ = 1*) and precisely one index case per household (*a_1_ = 1* and *a_2_ = 0*, or *a_1_ = 0* and *a_2_ = 1*). The assumption of no additional introductions from the outside into the household is particularly convenient, as it enabled us to obtain precise transmissibility estimates even with a study size of less than 50 households [23]. Because influenza virus infectious periods show only limited variation [24], the duration of the infectious period is assumed to be fixed (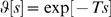). We have also considered models with exponentially distributed infectious periods, and arrived at the same conclusions (Table S2). For simplicity and given the lack of evidence to the contrary we further assume that the infectious period is type-independent, i.e. 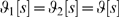. Without loss of generality we measure time in units equal to the duration of the infectious period and take 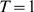 [22]. Further, we make the standard assumption that individuals make a fixed number of average contacts with each of the other persons in the household (i.e. we assume density dependent transmission) [25]. We have considered a model in which the average number of contacts is constant per unit of time (i.e. frequency dependent transmission) and arrived at essentially the same conclusions (Table S3).

With the above assumptions, the transmission rate from a type 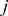 infected to a type 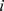 susceptible person (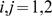) is given by 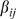. The secondary attack rate 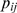 or probability that an infected type 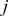 individual will infect a specific type 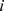 individual, given that the type 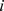 individual is initially susceptible and not infected by another person, is given by 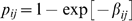 [5], [26]. Hence, the total number of type 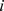 individuals that are potentially infected (i.e. assuming that they are not already infected by another individual) by an infected type 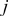 individual is binomially distributed with probability 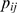 (i.e. the secondary attack rate) and number of trials given by the number of susceptible type 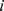 individuals.

The above model (labeled model E) is saturated and contains four parameters. To investigate to which extent simpler models are able to describe the data we consider a number of alternatives. First, we make a proportionate mixing assumption which states that the transmission parameter can be written as a product: 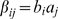 (model D). Specifically, we assume that transmission among type 1 individuals is represented by a parameter 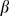, so that transmission from type 1 to type 2 individuals can be written as 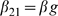, where 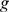 denotes the relative susceptibility of type 2 individuals. Likewise, the transmission rate from type *2* to type 1 individuals can be written as 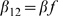, where 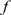 denotes the relative infectiousness of type 2 individuals. With this notation the transmission rate among type *2* individuals is given by 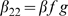. We simplified the proportionate mixing model further by assuming that type 1 and type *2* individuals are equally infectious (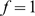)(model C), equally susceptible (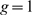)(model B), or equally susceptible and equally infectious (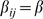)(model A). For completeness, to evaluate whether a transmission model is really needed to explain the data we also considered a model in which only the index case is infectious [25]. This model, however, is biologically implausible, had low statistical support in the analyses, and is therefore eliminated from further consideration.

Estimates of the parameters of interest are inferred from the household infection data using the method of maximum likelihood. Confidence intervals of the basic parameters, and confidence areas (models B and C) and volumes (model D) of parameter combinations are calculated on the basis of profile likelihoods [27]. Confidence intervals and confidence areas of the secondary attack rates, which are functions of the basic parameters, are determined by calculation of the range of values spanned of these functions on the confidence areas and volumes of the basic parameters. We compare nested models using likelihood ratio tests, and non-nested models using the small sample Akaike Information Criterion (AICc) [28]. We base our main results on the model with the lowest value of the AICc, and which is therefore most strongly supported by the data [28].

Although the moment of sampling of household contacts was probably close to ideal given that the generation interval of novel influenza A(H1N1) is approximately 2.2–3.2 days [5]–[6], [16]–[20], it is possible that some cases had been missed because sampling was performed too early or too late. The probability that a person does not have disease given a negative test result is called the negative predictive value of the test. To evaluate the robustness of our results to missed cases, we consider negative predictive values of the sampling and testing procedures ranging from 0.75 to 1, which corresponds to a sensitivity of detection of true influenza A(H1N1) cases ranging from 0.26 to 1. For a negative predictive value of *NPV*, we reclassified each uninfected person in the original data set (i.e., each person who tested negative) as infected with probability *1−NPV*. For each value of *NPV*, we repeated this procedure 1000 times to assemble 1000 alternative sets of true infection states that could have resulted in the observed data when infection was confirmed using a test with implied sensitivity
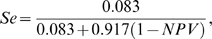
(2)where 9/109≈0.083 is the proportion of household contacts who tested positive for novel influenza A(H1N1).

## Results

**Figure 1:**
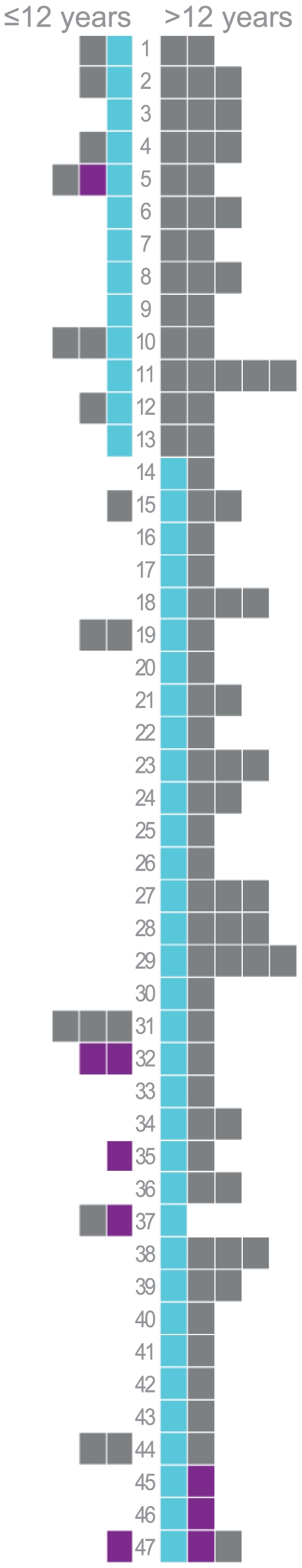
Overview of the household infection data. Household data were collected during the initial phase of the novel influenza A(H1N1) epidemic in the Netherlands. All household members, including the index case, were given oseltamivir upon detection of the index case. Each row represents a household and each square represents a person. Grey squares denote persons that are not infected, cyan squares correspond to index cases, and purple squares represent infected household members. Households are numbered 1 through 47. A distinction is made between younger persons (≤12 years of age, left of the household number) and older persons (>12 years of age, right of the household number).

In the period from 30 April 2009 to 22 June 2009 there were 47 households with a single virologically confirmed index case and one or more household members. All these households were included in the study. In 13 households the index case was 12 years or younger, and in the remaining 34 households the index case was older than 12 years. The average age of the index cases was 30 years (range: 3–69 years), which is comparable to the age distribution of infected cases in the initial phase of the pandemic when novel influenza A(H1N1) was still a notifiable disease in the Netherlands, and when most cases had a travel history to Mexico or the US.^20^ Infection of a total of 9 household members was confirmed in 7 out of 47 households; in all but one household the secondary infections occurred in households where the index case was older than 12 years. Of the nine household transmission events, six were to a younger person (≤12 years), and three to an older person (>12 years). Figure 1 gives an overview of the data.

**Figure 2:**
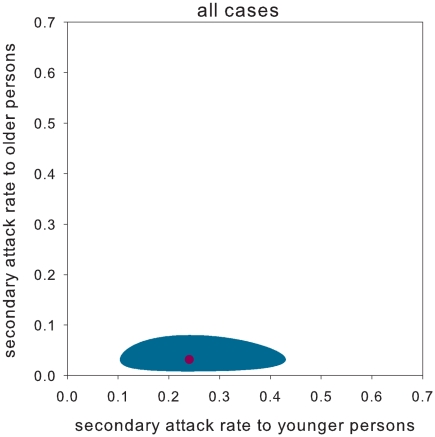
Estimated secondary attack rates for model C. Estimated secondary attack rates to younger (≤12 years of age) and older household members (>12 years of age). The maximum likelihood estimate is given by a red dot, and the 95% confidence region is indicated by the shaded area. The results are obtained using model C in Table 1. Note that the secondary attack rates are low, and that the entire 95% confidence region is below the identity line where younger and older persons are equally susceptible. The secondary attack rates to younger and older persons are determined by the basic transmission parameters through 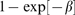 and 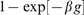, where 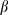 and 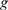 denote the transmission rate parameter among younger persons and the relative susceptibility of older persons.

The overall probability of transmission of infection from an infected person to an exposed household member is estimated at 0.075 (95%CI: 0.037–0.13) if younger and older household member are assumed to have similar levels of susceptibility and infectiousness (Table 1). Next we categorized index cases and their household members by age into those of 12 years and younger (who most likely received a lowered dose of oseltamivir) and those aged 13 years and older (who usually received the standard dose of oseltamivir). We detected a trend for a difference in infectiousness between younger and older cases, with older cases being more infectious than younger cases (model A vs B, 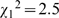, 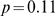), and compelling statistical evidence for a difference in susceptibility between younger and older persons, with older persons being less susceptible than younger ones (model A vs C, 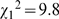, 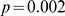). As a consequence, the probability of transmission to a younger person is substantially higher than that of transmission to an older person (Figure 2).

**Table 1:** Estimated secondary attack rates.

model	estimated secondary attack rate (95%CI)		number of parameters	AIC_c_	empirical support
A	y/o→y/o :	0.075 (0.037–0.13)	1	57.4	0.02 (weak)
B	y→y/o :	0.028 (0.0051–0.10)	2	57.1	0.02 (weak)
	o→y/o :	0.11 (0.049–0.20)			
C	y/o→y :	0.24 (0.10–0.43)	2	49.8	0.82 (substantial)
	y/o→o :	0.032 (0.0080–0.080)			
D	y→y :	0.092 (0.0056–0.34)	3	49.4	1 (strong)
	y→o :	0.011 (0.00057–0.056)			
	o→y :	0.35 (0.14–0.61)			
	o→o :	0.048 (0.012–0.12)			
E	y→y :	0.13 (0.0086–0.42)	4	51.0	0.45 (substantial)
	y→o :	0 (0–0.059)			
	o→y :	0.32 (0.12–0.59)			
	o→o :	0.057 (0.014–0.14)			

The secondary attack rates are defined as the person-to-person transmission probabilities over the complete infectious period of the infected person. Household members are categorized as younger (≤12 years of age, ‘y’) and older (>12 years of age, ‘o’). In model A the secondary attack rate does not depend on age, while in models B and C the secondary attack rates depend on the age of the infector (model B) or infected (model C). In models D and E the secondary attack rates depend both on the age of the infector and the age of the infected. In model E a separate transmission parameter is estimated for each transmission route, while in model D the secondary attack rates are based on the estimated relative susceptibility and infectiousness of older relative to younger persons. Calculation of the empirical support relative to the most likely model is based on the small sample Akaike's Information Criterion (AIC_c_).

**Figure 3:**
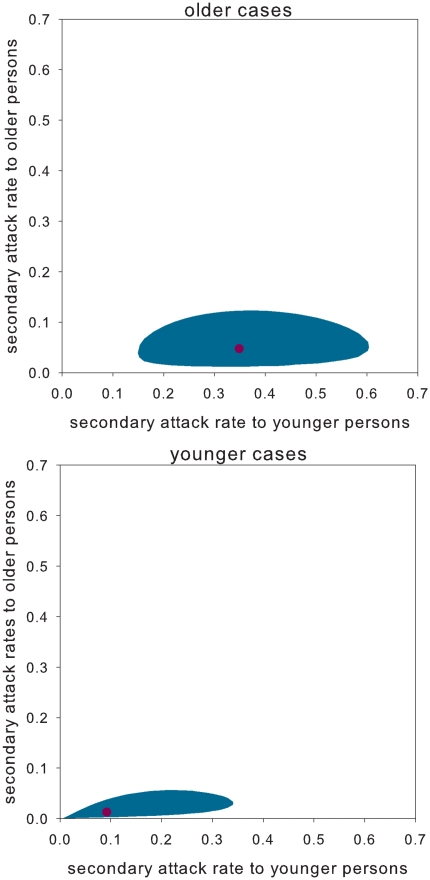
Estimated secondary attack rates for model D. Estimated secondary attack rates from older (>12 years of age) and younger household members (≤12 years of age). The maximum likelihood estimate is given by a red dot, and the 95% confidence regions are indicated by the shaded areas. The results are obtained using model D in Table 1. The top panel shows the results for transmission from older cases, and the bottom panel for transmission from younger cases. For younger cases the secondary attack rates to younger and older persons are given by 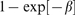 and 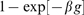, where 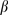 and 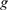 denote the transmission rate parameter among younger persons and the relative susceptibility of older persons. For older cases the secondary attack rates are 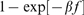 and 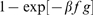, where 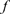 denotes the relative infectiousness of older persons.

The data also lend statistical support for simultaneous age-specific differences in infectiousness and susceptibility (model B vs D, 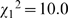, 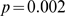; model C vs D, 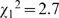, 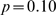), with older persons being less susceptible and more infectious than younger persons. There is no support for a statistical interaction between infectiousness and susceptibility (model D vs E, 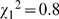, 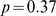). If we take the model with the highest statistical support (model D) the estimated probabilities of infection are 0.35 (95%CI: 0.14–0.61) from older to younger persons and 0.048 (95%CI: 0.012–0.12) among older persons. Because there is only one transmission event from a younger index case to another household member (Figure 1), the estimated probabilities of infection from younger persons to other household members are low. Overall, since the number of younger persons is substantially smaller than the number of older persons the estimated probabilities of transmission to younger persons are less precise than those of transmission to older persons (Figure 3). This is true if the case is an older person (Figure 3, top panel), and if the case if a younger person (Figure 3, bottom panel).

**Figure 4:**
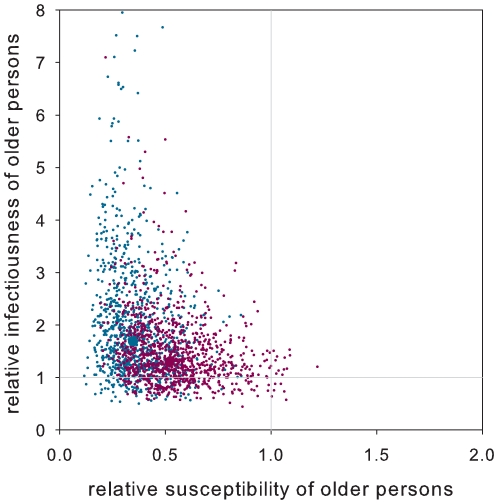
Age-specific susceptibility and infectiousness. Shown are the estimated susceptibility and infectiousness of older (>12 years of age) relative to younger (≤12 years of age) persons. Small dots indicate the parameter estimates for 1000 simulated datasets of actual infected states assuming negative predictive value of 90% (cyan; implied test sensitivity 47%) and 80% (purple; implied test sensitivity 30%). The results are obtained using model D in Table 1. Large dots represent median values of the parameter estimates. Irrespective of the negative predictive value, older persons are less susceptible than younger persons; if the negative predictive value is at least 95% (implied sensitivity: 64%), older cases are also more infectious than younger cases (cf. Table 2).

The testing procedures are not perfect, and it may be that a number of persons that are classified as uninfected were actually infected. This is mainly due to the fact that the timing of sampling is critical whether or not the test yields a positive result. Specifically, in view of the fact that the case finding procedure included taking swabs from all household members, and analysing these by a novel influenza A(H1N1) specific PCR it seems reasonable that the specificity of the testing procedure is close to 100%, while the sensitivity depends on the delay between the onset of symptoms of the index case and sampling of his/her household contacts. On average, this delay was 2.9 days, while the delay between the onset of symptoms of secondary cases and sampling of secondary case was 0.6 days (see Methods for details). Given that the generation interval of novel influenza A(H1N1) is probably in the range 2–4 days [5]–[6], [16]–[20], we believe that the moment of sampling of household contacts may have been close to optimal. Nevertheless, it is possible that we may have missed a couple of cases, and we have reanalysed the data while explicitly accounting for the possibility that test sensitivity is not 100% (see Methods). The analyses reveal that our findings are robust, and that even if the negative predictive value is as low as 80% (implying a test sensitivity of just 31%), there is evidence for older persons being less susceptible to infection than younger persons, while there is evidence for younger cases to be less infectious than older cases if negative predictive value is at least 95% (implying a test sensitivity of at least 64%)(Table 2, Figure 4).

**Table 2:** Impact of test sensitivity on the parameter estimates.

negative predictive value^1^	implied test sensitivity^1^	relative susceptibility of older persons (95%CI)	relative infectiousness of older persons (95%CI)
100%	100%	0.11^2^	4.4^1^
95%	64%	0.24 (0.12–0.44)	2.2 (0.97–7.8)
90%	47%	0.35 (0.17–0.63)	1.7 (0.73–5.1)
85%	38%	0.45 (0.21–0.82)	1.5 (0.69–4.1)
80%	31%	0.52 (0.27–0.98)	1.3 (0.70–3.2)
75%	26%	0.59 (0.31–1.1)	1.3 (0.68–2.8)

Overview of the impact of imperfect test sensitivity on the estimated susceptibility and infectiousness of older persons (>12 years of age) relative to younger persons (≤12 years of age). The results are obtained using model D in Table 1. For each assumed value of the negative predictive value we give the medians of the parameter estimates of 1000 simulated datasets with 95% bootstrap confidence intervals (between brackets).

1 : see equation (2) for the calculation of implied test sensitivity.

2 : 95% confidence intervals calculated using the profile likelihood are (0.024–0.43) for relative susceptibility and (0.77–83) for relative infectiousness.

Throughout we have categorized persons using an age classification of ≤12 years versus >12 years, which coincides with the age above which a standard dose of oseltamivir is recommended. We also investigated the data using an age classification of ≤18 years versus >18 years. Using this age classification there were 52 younger and 104 older persons in the 47 households. Of the 47 households, there were 18 households with a younger index case, and the remaining 29 households had an older index case. There were 6 younger secondary infections and 3 older secondary infections, which coincides with our earlier age classification. Again, we find statistical support for age-dependent susceptibility and infectiousness, with older household members being less susceptible and more infectious than younger household members (Table 3). In comparison with the analyses with the original age classification the estimated secondary attack rates to younger persons are lower, while the precision of the estimates increase. This is due to the fact that the total number of younger persons is higher than in the analyses with an age cut-off of 13 years (52 versus 35). On the other hand, estimates of the secondary attack rate from older persons to older persons increase, while the precision of the estimates decreases.

**Table 3:** Estimated secondary attack rates using an alternative age classification.

model	estimated secondary attack rate (95%CI)		number of parameters	AIC_c_	empirical support
A	y/o→y/o :	0.075 (0.037–0.13)	1	57.4	0.05 (weak)
B	y→y/o :	0.019 (0.0054–0.078)	2	53.7	0.30 (substantial)
	o→y/o :	0.15 (0.067–0.26)			
C	y/o→y :	0.16 (0.068–0.30)	2	54.3	0.22 (substantial)
	y/o→o :	0.036 (0.0092–0.092)			
D	y→y :	0.043 (0.0025–0.18)	3	51.3	1 (strong)
	y→o :	0.010 (0.00053–0.051)			
	o→y :	0.28 (0.12–0.50)			
	o→o :	0.073 (0.018–0.19)			
E	y→y :	0.071 (0.0044–0.26)	4	52.6	0.52 (substantial)
	y→o :	0 (0–0.045)			
	o→y :	0.26 (0.091–0.48)			
	o→o :	0.086 (0.022–0.21)			

Estimated secondary attack rates using an alternative age classification of ≤18 versus >18 years of age. The lay out and model scenarios are as in Table 1. Here, too, we find low secondary attack rates and strong statistical support for age-dependent susceptibility and infectiousness.

To further test the robustness of the results we have analyzed model scenarios with exponentially distributed infectious periods (Table S2) and with frequency dependent instead of density dependent transmission (Table S3). The frequency dependent transmission models fit the data better than the density dependent transmission models, suggesting that secondary attack rates may be lower in larger households than in smaller households. Overall, however, the analyses indicate that our main conclusions of limited overall transmission, and higher susceptibility of younger persons are robust, and are also found in alternative model scenarios.

## Discussion

Based on detailed early data from the Netherlands we have estimated transmission probabilities of novel influenza A(H1N1) in human households where the index case and household members use the antiviral drug oseltamivir. In this setting the overall transmission levels are low (0.075, 95%CI:0.037–0.13), children are substantially more susceptible to infection than adolescents and adults, and the highest secondary attack rates are found for transmission from an older case to a younger person. Specifically, the estimated secondary attack rate from an older case to a younger person is 0.35 (95%CI: 0.14–0.61) when using an age classification of ≤12 versus >12 years, and 0.28 (95%CI: 0.12–0.50) using an age classification of ≤18 versus >18 years.

Earlier studies that focused on seasonal influenza A have found similar household infection probabilities, and similar differences between children/adolescents and adults. For instance, analysis of data from the 1977–1978 H3N2 epidemic indicate that the household infection probabilities are approximately 0.25 for transmission to a child/adolescent and 0.10 for transmission to an adult [11], [13]–[14], while analyses of two trials aimed at estimation of the efficacy of prophylactic use of oseltamivir and that were carried out in 1988–1999 and 2000–2001 yielded estimated child-to-child and adult-to-adult secondary attack rates of 0.15 and 0.086, respectively [26], [29]–[31].

A number of recent household studies have investigated the transmissibility of novel influenza A(H1N1) in Japan, The United Kingdom, and the United States [4]–[5], [15]–[16]. Overall, the percentage of infected non-index cases ranged from less than 5% to more than 25%, with percentages infected of 8–11% and 13% in the largest studies [4], [16]. These figures correspond reasonably well with our finding of 9/109 = 8.3% infected non-index cases. Our results also confirm the earlier finding that younger persons are substantially more susceptible to infection than older persons [4], [15]–[16]. Interestingly, our tailored analyses suggest (non-significantly) that older cases may be more infectious than younger cases, which had not been observed before.

We have quantified novel influenza A(H1N1) household transmission in a setting where all persons were given the antiviral drug oseltamivir. Because antiviral drugs were given to all persons this makes it impossible to estimate antiviral efficacy for susceptibility and infectiousness. However, assuming that the efficacy of oseltamivir against novel influenza A(H1N1) is not different from its efficacy against seasonal influenza A viruses, we could use published antiviral efficacy estimates to gauge what the transmission probabilities would have been in case that no antiviral drugs were given [9]. Arguing along these lines and focusing on the antiviral efficacy for susceptibility, one can obtain rough estimates for what the secondary attack rates would have been if no antiviral drugs were used. For instance, if we focus on model D in Table 1 and make the plausible assumption that antiviral efficacy for susceptibility is 0.50 [9], the transmission rates would increase from an estimated 0.097 (y→y), 0.011 (y→o), 0.43 (o→y), and 0.049 (o→o) per infectious period (Table 1) to 0.19 (y→y), 0.022 (y→o), 0.86 (o→y), and 0.098 (o→o) per infectious period. This in turn translates into secondary attack rates of 0.18 (y→y), 0.022 (y→o), 0.58 (o→y), and 0.093 (o→o). Hence, if the estimated secondary attack rates are small the secondary attack rates without antiviral drugs increase approximately by a factor 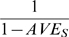, and by less if the estimated secondary attack rates are already high. Although the projected secondary attack rates of 0.18, 0.022, 0.58, and 0.093 in a situation where no antiviral drugs would have been given are substantially higher than the original estimates of Table 1, they are still fairly low except for the older-to-younger transmission route.

Our finding of limited overall household transmission for a novel influenza virus against which little pre-existing immunity exists could be due to the fact that antiviral drugs were given to all household members upon detection of infection. In fact, it is known that antiviral drugs are effective both in preventing infection as well as mitigating the severity of infection, with the former probably more important than the latter [9], [29]–[33]. An alternative explanation is that influenza transmission in general is suppressed in summer season. It is known that influenza epidemics in temperate regions are highly seasonal, and that this seasonality may be modulated by environmental conditions such as temperature and humidity [34]–[35]. This explanation is not fully satisfactory, however, as not all estimated transmission rates are low, in particular the rate of transmission from older to younger persons.

Overall, our results of limited overall transmission and higher susceptibility of younger persons are robust. In fact, we obtain similar results in a variety of alternative analyses, e.g., using a different age classification (Table 3), allowing for misclassification due to imperfect test sensitivity (Table 2, Figure 3), using a highly variable distribution of the infectious period (Table S2), or assuming frequency dependent instead of density dependent transmission (Table S3). It is of note that some of the alternative analyses give a slightly better fit to the data than our default analyses. For instance, a model with a highly variable exponentially distributed infectious period (Table S2) fits the data slightly better than a model with a fixed infectious period (Table 1). This does not reflect current understanding of the epidemiology of influenza, however, as it is known that influenza A infections show only very limited variation in the period of shedding [24]. Interestingly, a scenario with frequency dependent transmission, i.e. which assumes that a person makes a fixed number of contacts per unit of time, also gives a better fit to the data than our conventional density dependent transmission model which assumes that a person makes a fixed number of contacts *with each of the other household members* per unit of time [25]. Since the number of households and the variation in household sizes in our study (2–6) is limited it is difficult to judge the relevance of this finding. It could, however, have important implications as the overall attack rates are expected to increase with increasing household size in a density dependent model but remain approximately constant in a frequency dependent transmission model.

We envisage four possible explanations for the observed differences in attack rates between younger and older persons. First, differences in susceptibility may be related to antiviral treatment [9], [29]–[33]. In the Netherlands, children under 10–12 years of age or weighing less than 40kg are prescribed a lower dose of oseltamivir than adults (75mg per 12h in adults to 30/45mg per 12h in children 1–6 years old), and this could conceivably have a negative impact on the effectiveness of the drug. Second, compliance with antiviral treatment is often imperfect, especially in children, because of the side effects associated with the use of oseltamivir, while the protective effect in children is possibly smaller than in adults [33]. Third, the nature of contacts between children and adults could be such that the virus is more easily transmitted from an adult to a child than the other way around. In this case, a contact that is sufficient for transmission from an adult to a child may not be sufficient for transmission from child to adult, and case contacts are said to be asymmetrical [36]. Fourth, children may be intrinsically more susceptible to infection than adults. Possible reasons for such differences include pre-existing immunity in adults. This is an attractive explanation which is consistent with observations and estimates for seasonal influenza A [8], [12]. Regardless of the precise explanation for the observed difference our results suggest that preventive household measures can be made more effective by focusing specifically on the adult-to-child transmission route.

## Supporting Information

Table S1 Likelihood contributions of the individual households. For each of the 47 households the infectious period is assumed to be of fixed duration while using the standard age classification (< = 12 versus >12 years of age).
(0.03 MB PDF)

Table S2 Estimated secondary attack rates using an exponentially distributed infectious period. Household members are categorized as younger (< = 12 years of age, ‘y’) and older (>12 years of age, ‘o’). See Table 1 for overview of model scenarios.
(0.04 MB PDF)

Table S3 Estimated transmission rate parameters assuming frequency dependent transmission. Household members are categorized as younger (< = 12 years of age, ‘y’) and older (>12 years of age, ‘o’). See Table 1 for overview of model scenarios.
(0.05 MB PDF)
