# Supplementary material for: Transmission of Novel Influenza A(H1N1) in Households with Post-Exposure Antiviral Prophylaxis
Source: PLoS One. 2010 Jul 7;5(7):e11442. doi: 10.1371/journal.pone.0011442 (PMC2898802; doi:10.1371/journal.pone.0011442)
Supplement: Table S1 — Likelihood contributions of the individual households. For each of the 47 households the infectious period is assumed to be of fixed duration while using the standard age classification (< = 12 versus >12 years of age). (0.03 MB PDF) [file pone.0011442.s001.pdf]

Table S1. Likelihood contributions of the 47 households when assuming a fixed infectious period, and using an age classification of ≤12 versus > 12 years of age.

household nr	likelihood contribution
1	$e^{-\beta_{11}-2\beta_{21}}$
2	$e^{-\beta_{11}-3\beta_{21}}$
3	$e^{-3\beta_{21}}$
4	$e^{-\beta_{11}-3\beta_{21}}$
5	$2e^{-3\beta_{11}-4\beta_{21}}\left(e^{\beta_{11}}-1\right)$
6	$e^{-3\beta_{21}}$
7	$e^{-2\beta_{21}}$
8	$e^{-3\beta_{21}}$
9	$e^{-2\beta_{21}}$
10	$e^{-2(\beta_{11}+\beta_{21})}$
11	$e^{-5\beta_{21}}$
12	$e^{-\beta_{11}-2\beta_{21}}$
13	$e^{-2\beta_{21}}$
14	$e^{-\beta_{22}}$
15	$e^{-\beta_{12}-2\beta_{22}}$
16	$e^{-\beta_{22}}$
17	$e^{-\beta_{22}}$
18	$e^{-3\beta_{22}}$
19	$e^{-2\beta_{12}-\beta_{22}}$
20	$e^{-\beta_{22}}$
21	$e^{-2\beta_{22}}$
22	$e^{-\beta_{22}}$
23	$e^{-3\beta_{22}}$
24	$e^{-2\beta_{22}}$
25	$e^{-\beta_{22}}$
26	$e^{-\beta_{22}}$
27	$e^{-3\beta_{22}}$
28	$e^{-3\beta_{22}}$
29	$e^{-4\beta_{22}}$
30	$e^{-\beta_{22}}$
31	$e^{-3\beta_{12}-\beta_{22}}$
32	$e^{-\beta_{11}-2\beta_{12}-2\beta_{21}-\beta_{22}}\left(e^{\beta_{12}}-1\right)\left(e^{\beta_{11}}+e^{\beta_{11}+\beta_{12}}-2\right)$
33	$e^{-\beta_{22}}$
34	$e^{-2\beta_{22}}$
35	$e^{-\beta_{12}-\beta_{21}-\beta_{22}}\left(e^{\beta_{12}}-1\right)$
36	$e^{-2\beta_{22}}$
37	$2e^{-\beta_{11}-2\beta_{12}}\left(e^{\beta_{12}}-1\right)$
38	$e^{-3\beta_{22}}$
39	$e^{-2\beta_{22}}$
40	$e^{-\beta_{22}}$
41	$e^{-\beta_{22}}$
42	$e^{-\beta_{22}}$
43	$e^{-\beta_{22}}$
44	$e^{-2\beta_{12}-\beta_{22}}$
45	$1-e^{-\beta_{22}}$
46	$1-e^{-\beta_{22}}$
47	$2e^{-2\beta_{12}-2\beta_{21}-3\beta_{22}}\left(e^{\beta_{12}}-1\right)\left(e^{\beta_{21}+\beta_{22}}+e^{\beta_{12}+\beta_{21}+\beta_{22}}-e^{\beta_{12}}-e^{\beta_{21}}\right)$
