# Supplementary material for: Transmission of Novel Influenza A(H1N1) in Households with Post-Exposure Antiviral Prophylaxis
Source: PLoS One. 2010 Jul 7;5(7):e11442. doi: 10.1371/journal.pone.0011442 (PMC2898802; doi:10.1371/journal.pone.0011442)
Supplement: Table S2 — Estimated secondary attack rates using an exponentially distributed infectious period. Household members are categorized as younger (< = 12 years of age, ‘y’) and older (>12 years of age, ‘o’). See Table 1 for overview of model scenarios. (0.04 MB PDF) [file pone.0011442.s002.pdf]

Table S2. Estimated secondary attack rates using an exponentially distributed infectious period

model	estimated secondary attack rate (95%CI)	number of parameters	AIC_c	empirical support
A	y/o→y/o : 0.081 (0.038-0.15)	1	56.7	0.01 (weak)
B	y→y/o : 0.025 (0.0014-0.11)<br>o→y/o : 0.12 (0.053-0.25)	2	56.0	0.02 (weak)
C	y/o→y : 0.34 (0.11-0.58)<br>y/o→o : 0.035 (0.0083-0.090)	2	49.2	0.50 (substantial)
D	y→y : 0.086 (0.0048-0.39)<br>y→o : 0.0073 (0.0032-0.051)<br>o→y : 0.49 (0.18-0.91)<br>o→o : 0.054 (0.013-0.15)	3	47.8	1 (strong)
E	y→y : 0.11 (0.0065-0.48)<br>y→o : 0 (0-0.061)<br>o→y : 0.45 (0.14-0.89)<br>o→o : 0.060 (0.015-0.16)	4	49.6	0.41 (substantial)

Household members are categorized as younger (≤12 years of age, 'y') and older (>12 years of age, 'o'). The lay out and model scenarios are as in Table 1. Here, too, we find low transmission probabilities and strong statistical support for age-dependent susceptibility and infectiousness.
