# Supplementary material for: Transmission of Novel Influenza A(H1N1) in Households with Post-Exposure Antiviral Prophylaxis
Source: PLoS One. 2010 Jul 7;5(7):e11442. doi: 10.1371/journal.pone.0011442 (PMC2898802; doi:10.1371/journal.pone.0011442)
Supplement: Table S3 — Estimated transmission rate parameters assuming frequency dependent transmission. Household members are categorized as younger (< = 12 years of age, ‘y’) and older (>12 years of age, ‘o’). See Table 1 for overview of model scenarios. (0.05 MB PDF) [file pone.0011442.s003.pdf]

Table S3. Overview of transmission rate parameters for different household transmission models when assuming frequency dependent transmission.

model	estimated transmission rate parameter (95%CI)	number of parameters	AIC_c	empirical support
A	y/o→y/o : 0.28 (0.13-0.50)	1	56.0	<0.01 (negligible)
B	y→y/o : 0.13 (0.0083-0.40)<br>o→y/o : 0.37 (0.14-0.51)	2	56.9	<0.01 (negligible)
C	y/o→y : 1.2 (0.45-2.4)<br>y/o→o : 0.11 (0.028-0.29)	2	46.7	1 (strong)
D	y→y : 0.48 (0.029-2.0)<br>y→o : 0.043 (0.0022-0.23)<br>o→y : 1.7 (0.60-3.7)<br>o→o : 0.15 (0.036-0.40)	3	47.2	0.78 (substantial)
E	y→y : 0.64 (0.040-2.5)<br>y→o : 0 (0-0.27)<br>o→y : 1.5 (0.48-3.6)<br>o→o : 0.17 (0.042-0.45)	4	48.9	0.33 (substantial)

Household members are categorized as younger (≤12 years of age, ‘y’) and older (>12 years of age, ‘o‘). The household size specific secondary attack rates $p_{ij}(N)$ are determined by the transmission rate parameters β_{ij} through the transformation $p_{ij}(N) = 1 - \exp\left[-\frac{\beta_{ij}}{N}\right]$, where N denotes household size. See Table 1 for overview of model scenarios.
